# *Symphytum* Species: A Comprehensive Review on Chemical Composition, Food Applications and Phytopharmacology

**DOI:** 10.3390/molecules24122272

**Published:** 2019-06-18

**Authors:** Bahare Salehi, Farukh Sharopov, Tugba Boyunegmez Tumer, Adem Ozleyen, Celia Rodríguez-Pérez, Shahira M. Ezzat, Elena Azzini, Tahereh Hosseinabadi, Monica Butnariu, Ioan Sarac, Cristian Bostan, Krishnendu Acharya, Surjit Sen, Kadriye Nur Kasapoglu, Ceren Daşkaya-Dikmen, Beraat Özçelik, Navid Baghalpour, Javad Sharifi-Rad, Patrick Valere Tsouh Fokou, William C. Cho, Natália Martins

**Affiliations:** 1 Student Research Committee, School of Medicine, Bam University of Medical Sciences, Bam 44340847, Iran; bahar.salehi007@gmail.com; 2 Department of Pharmaceutical Technology, Avicenna Tajik State Medical University, Rudaki 139, Dushanbe 734003, Tajikistan; farukhsharopov@gmail.com; 3 Department of Molecular Biology and Genetics, Faculty of Arts and Science, Canakkale Onsekiz Mart University, 17020 Canakkale, Turkey; tumertb@gmail.com; 4 Graduate Program of Biomolecular Sciences, Institute of Natural and Applied Sciences, Canakkale Onsekiz Mart University, 17020 Canakkale, Turkey; ademozleyen@gmail.com; 5 Department of Nutrition and Food Science, University of Granada, Campus of Cartuja, E-18071 Granada, Spain; celiarp@ugr.es; 6 Institute of Nutrition and Food Technology (INYTA) ‘José Mataix’, Biomedical Research Centre, University of Granada, Avenida del Conocimiento s/n, E-18071 Granada, Spain; 7 Pharmacognosy Department, Faculty of Pharmacy, Cairo University, Kasr El-Ainy Street, Cairo 11562, Egypt; shahira.ezzat@pharma.cu.edu.eg; 8 Department of Pharmacognosy, Faculty of Pharmacy, October University for Modern Science and Arts (MSA), 6th October 12566 Egypt; 9 Centre for Research on Food and Nutrition, Council for Agricultural Research and Economics, 546-00178 Rome, Italy; elena.azzini@crea.gov.it; 10 Department of Pharmacognosy and Biotechnology, School of Pharmacy, Shahid Beheshti University of Medical Sciences, Tehran 11369, Iran; hosseinabadi.t@gmail.com; 11 Banat’s University of Agricultural Sciences and Veterinary Medicine “King Michael I of Romania” from Timisoara, 300645 Calea Aradului 119, Timis, Romania; monicabutnariu@yahoo.com (M.B.); ionutsarac@yahoo.com (I.S.); cristian.bostan.tm@gmail.com (C.B.); 12 Molecular and Applied Mycology and Plant Pathology Laboratory, Department of Botany, University of Calcutta, Kolkata 700019, India; krish_paper@yahoo.com (K.A.); surjitsen09@gmail.com (S.S.); 13 Department of Botany, Fakir Chand College, Diamond Harbour, West Bengal 743331, India; 14 Istanbul Technical University, Chemical and Metallurgical Engineering Faculty, Food Engineering Department, Ayazağa Campus, Maslak, 34469 Istanbul, Turkey; kasapogluk@itu.edu.tr (K.N.K.); cerenddikmen@gmail.com (C.D.-D.); ozcelik@itu.edu.tr (B.Ö.); 15 Istanbul Gedik University, Department of Gastronomy and Culinary Arts, Kartal, 34876 Istanbul, Turkey; 16 Bioactive Research & Innovation Food Manufac. Indust. Trade Ltd., Katar Street, Teknokent ARI-3, B110, Sarıyer, 34467 Istanbul, Turkey; 17 Phytochemistry Research Center, Shahid Beheshti University of Medical Sciences, Tehran 11369, Iran; navid.bp1994@gmail.com; 18 Zabol Medicinal Plants Research Center, Zabol University of Medical Sciences, Zabol 61615-585, Iran; 19 Antimicrobial and Biocontrol Agents Unit, Department of Biochemistry, Faculty of Science, University of Yaounde 1, Ngoa Ekelle, Annex Fac. Sci, Yaounde 812, Cameroon; 20 Department of Clinical Oncology, Queen Elizabeth Hospital, 30 Gascoigne Road, Hong Kong, China; 21 Faculty of Medicine, University of Porto, Alameda Prof. Hernâni Monteiro, 4200-319 Porto, Portugal; 22 Institute for Research and Innovation in Health (i3S), University of Porto, 4200-135 Porto, Portugal

**Keywords:** *Symphytum* species, phytochemistry, biological activity, clinical trials

## Abstract

*Symphytum* species belongs to the Boraginaceae family and have been used for centuries for bone breakages, sprains and rheumatism, liver problems, gastritis, ulcers, skin problems, joint pain and contusions, wounds, gout, hematomas and thrombophlebitis. Considering the innumerable potentialities of the *Symphytum* species and their widespread use in the world, it is extremely important to provide data compiling the available literature to identify the areas of intense research and the main gaps in order to design future studies. The present review aims at summarizing the main data on the therapeutic indications of the *Symphytum* species based on the current evidence, also emphasizing data on both the efficacy and adverse effects. The present review was carried out by consulting PubMed (Medline), Web of Science, Embase, Scopus, Cochrane Database, Science Direct and Google Scholar (as a search engine) databases to retrieve the most updated articles on this topic. All articles were carefully analyzed by the authors to assess their strengths and weaknesses, and to select the most useful ones for the purpose of review, prioritizing articles published from 1956 to 2018. The pharmacological effects of the *Symphytum* species are attributed to several chemical compounds, among them allantoin, phenolic compounds, glycopeptides, polysaccharides and some toxic pyrrolizidine alkaloids. Not less important to highlight are the risks associated with its use. In fact, there is increasing consumption of over-the-counter drugs, which when associated with conventional drugs can cause serious and even fatal adverse events. Although clinical trials sustain the folk topical application of *Symphytum* species in musculoskeletal and blunt injuries, with minor adverse effects, its antimicrobial potency was still poorly investigated. Further studies are needed to assess the antimicrobial spectrum of *Symphytum* species and to characterize the active molecules both in vitro and in vivo.

## 1. Introduction

*Symphytum* species, also known as comfrey, belong to the Boraginaceae family and include around 35 species, including *Symphytum officinale* L., *Symphytum tuberosum* L., *Symphytum x uplandicum* Nyman, *Symphytum asperum* Lepech and *Symphytum caucasicum* Bieb [1,2]. *S. officinale* often called herb comfrey, is the most commonly used species [3,4]. Other species, like *Symphytum asperum* (prickly comfrey) and *S. × uplandicum* (Russian comfrey, syn. *Symphytum peregrinum*) have also been used [3].

Comfrey has been recommended for external and internal use as a botanical preparation for more than 2000 years [5]. Worldwide, ethnographic studies have reported the external use of both *S. officinale* and *S. tuberosum* for osteoarticular disturbances in Navarra; the tea for hepatic disturbances, and as an internal therapy for rheumatism in Mexico; gastritis and ulcers in Brazil and skin problems in USA and roots (tea, alcoholic extract or ointment) for osteoarticular pain in Lithuania or as tonic in Jamaica [6]. Comfrey extracts, ointments or compress pastes are applied externally, while the crude plant parts (leaf, herb or root) have been traditionally used for musculoskeletal disorders, wounds, gout, hematomas and thrombophlebitis [3,4,5].

Comfrey diversity, bioactivities and related efficacies is a manifestation of its complex effects and a source of opportunities for researchers from various disciplines, among them pharmacy [3]. Pharmacological effects of comfrey extracts are attributed to several chemical compounds. Allantoin has been claimed as the active ingredient of comfrey, which is responsible for triggering cell division and would healing, also promoting conjunctive tissue, bone and cartilage growth [7,8]. *S. officinale* root extract contains allantoin and phenolic acids (e.g., rosmarinic, *p*-hydroxybenzoic, caffeic, chlorogenic and *p*-coumaric acids), and displays remarkable antioxidant effects, besides to exert a positive impact in human skin fibroblasts [9]. Barbakadze et al. indicated that *S. asperum* and *S. caucasicum* leaves had an anticomplement and antioxidant potential [10]. Moreover, the strong antioxidant effects have been linked to polysaccharides, having a uronic acid group in the comfrey root [11]. Enzyme-ultrasonic assistance technology has been reported as a great strategy to isolate polysaccharides from the comfrey root, that exhibit notable α-glucosidase inhibition activity [11].

Besides its folk medicinal applications, comfrey also contains some molecules (e.g., symphytine, echimidine, pyrrolizidine alkaloids) that have been related to hepatic cancers and related toxicity, pneumotoxicity, genotoxicity and carcinogenicity in animal and human studies [12,13,14,15,16]. In humans, comfrey consumption has been associated with individual cases of hepatotoxic reactions: Liver fibrosis, portal hypertension and veno-occlusive diseases [17]. Conversely, no adverse events have been reported when externally used; indeed, pharmacokinetic studies have reported a very low cutaneous absorption [16]. Hence, its internal application is not recommended by regulatory agencies and international health organizations [12]. However, in the UK, comfrey is only available over-the-counter for external applications, and when prescribed by qualified medical herbalists, for internal or external use. In the USA, Canada and some European countries, such as Germany, Denmark and Austria, comfrey use is under restriction. Commission E recommends a restriction in its use to 4–6 weeks/year [18].

*S. officinale* leaf extract loaded silver nanoparticles has been suggested to be used as an agent against skin photoaging due to its photoprotective potential [19]. Comfrey leaves-derived extracts also displayed a great inhibition on fungal pathogens germination and modulate the mechanisms of plant defense [20]. The studies on comfrey extracts reported that this plant could be used in the management of pests and diseases [21]. In addition to the antifungal activity of comfrey, its antibacterial activity against the bacteria causing bovine mastitis has also been reported [22]. Indeed, Oliveira et al. introduced *S. officinale* in the broiler diet and found that it could be used as a growth-promoting antibiotic for feeding broiler [23].

Other studies performed in humans have also examined the comfrey potential for back pain, wounds and arthritis. Comfrey has also been proposed as an alternative solution for treating skin conditions [2], there even being found a moderate effectiveness of comfrey cream in osteoarthritis [6]. However, the adverse events related to the use of this plant were not assessed in human studies so far; indeed, the safety assessments were mostly performed in single case reports and obtained from animal studies using high doses. Therefore, its safe assessment is absent using biochemical markers and epidemiological methods in humans [3].

In this sense, this manuscript aims to provide a detailed review of the most recent studies on comfrey, including their habitat and cultivation conditions, phytochemical composition and to discuss their antimicrobial, antioxidant, anticancer activities through in vitro and in vivo data, and clinical effectiveness.

## 2. *Symphytum* Species: Habitat and Cultivation

### 2.1. Habitat

*Symphytum* species are mesophytic, perennial herbs belonging to the Boraginaceae family. This genus comprises approximately 40 species, among them, 14 species are reported from Europe mainly in peninsular Italy [24,25] and around 18 species are reported from Turkey [26]. Native from Europe and Asia [27], this genus thrives well in moist, cool places on river banks and streams, ditches of roads and damp grasslands.

### 2.2. Cultivation

#### 2.2.1. Optimum Growing Parameters

*Symphytum* species grows in temperate and subtropical regions. It adapted well and gives a maximum yield under a cold condition with full sunlight [28]. Due to its deep root system, the plant is drought tolerant. The plant is also very frost resistant. It is a fast-growing plant. If weeds and soil fertility is properly maintained the crop should last indefinitely.

#### 2.2.2. Soil

Although easily adaptable to any soil, comfrey prefers moist, fertile and rich in organic matter [29,30]. The plant grows well in sandy looms with adequate nutrients mainly if nitrogen is present in the soil [28], and even if the soil is amended with compost material [31]. The ideal soil pH ranges from 6.0 to 7.0.

#### 2.2.3. Propagation

The crop is generally propagated through transplants, crown cuttings with buds and root cuttings as the rate of seed production are very low [32]. High yield is recorded from transplant and least from root cutting during the first year of propagation, but after that, there is no difference in yield regardless of the propagation method. Propagation through root cuttings is less expensive and a frequently used method. Cutting size varies from 1½ to 6 inches long and from ¼ to ¾ inches in diameter are common. Root cutting with comparatively smaller size generate plants, but longer cutting establish and emerge faster. It has been reported that successful in vitro propagation from root explants produce a high amount of clones, more quickly than by the conventional vegetative propagation [33].

#### 2.2.4. Planting

The best time for comfrey planting is April, but the crop can be planted throughout the growing season. Teynor et al. reported that a root cutting should be planted before September, while early October is suitable for both transplant or crown cuttings [28]. Cuttings should be treated with cold water for an adequate time before planting [34]. Planting depth varies with soil texture and soil moisture content. Four inches depth is common, but up to two inches depth is also practiced with an adequate irrigation system. Comfrey is generally planted in a checkerboard pattern at rows of 3 to 4 ft. apart to allow cross cultivation for weed control. It has been found that closer row spacing (about 30 inches) gives better yield, but the cost of cutting becomes higher [28,34].

#### 2.2.5. Maturing

Comfrey contains a high amount of proteins and the plant obtains all its nitrogen from the soil, so it is necessary to amend nitrogen fertilizer or compost by broadcasting or side dressing [35]. Older plantings that develop lighter green color leaves require broadcasting of nitrogen fertilizer. Barnyard manure is an excellent fertilizer for comfrey. Sometimes powdered limestone and rock phosphate can be added if the soil is too acidic. The crop can be benefited by the addition of animal manure or litter of poultry, considered nitrogen-rich sources.

#### 2.2.6. Weed-Pest Management

Due to the fast and dance growth habit of comfrey, it is an excellent weed competitor. Weeds may establish between the comfrey clumps under a multiple-cut harvesting practice. Rototilling between plants is an effective method of controlling weeds [28]. The crop has usually been grown without herbicides. It has been reported that comfrey is tolerant to 2,4-D and 2,4,5-T and susceptible to atrazine, sodium chloride and ammonium sulfamate [34]. Diseases have not been a serious problem in this crop. It is reported that the rust fungus, *Melampsorella symphyti*, is associated with comfrey roots, reducing its yield [28]. A rare association of alfalfa mosaic virus (AMV) was reported in comfrey, showing yellow spots, rings and chlorotic line patterns [36]. Insect pests are also not reported much in this crop. Foliar nematodes (*Aphelenchoides fragariae* and *A. ritzemabosi*) are associated with a crop yield reduction [37,38].

#### 2.2.7. Harvesting

Mature comfrey can be harvested 4–5 times/year. When the seasonal conditions are suitable, usually during mid-spring and the crop reaches a height of about 24 inches, they are ready for harvesting. The optimum period to cut the crop is just before flowering as it is the most effective in terms of the nutrient that it produces [34].

## 3. Phytochemical Composition

**Figure 1:**
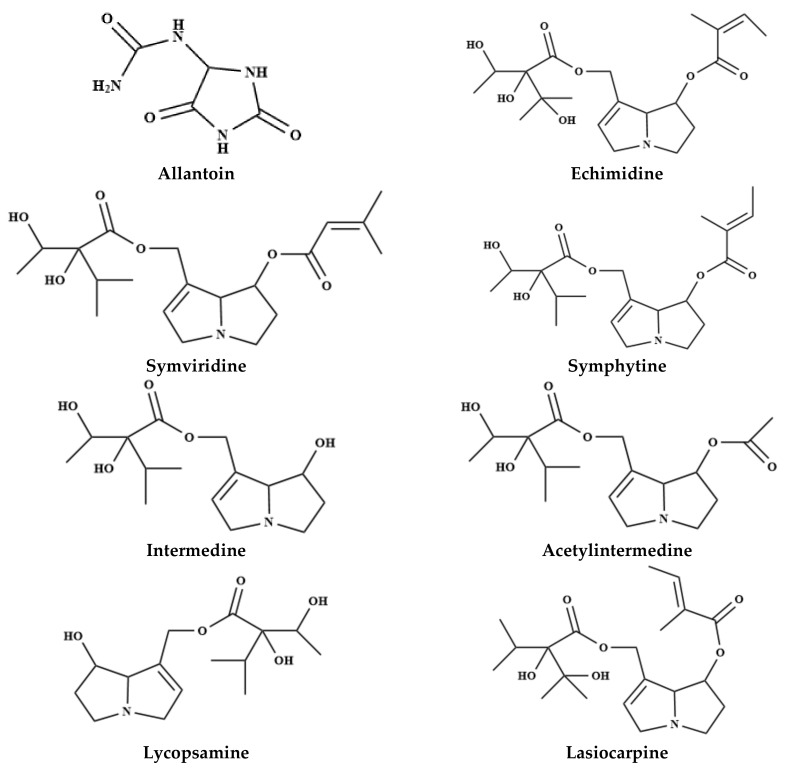
Chemical structure of the main heterocyclic compounds of the *Symphytum* species.

The several therapeutic properties attributed to comfrey came from the different main constituents present in the root together with the leaves and the flowering tops of the plants. Their therapeutic properties include anti-inflammatory, analgesic, granulation promoting and anti-exudative effects. Indeed, comfrey contains a wide variety of critically important bioactive constituents, including allantoin, RA, triterpene saponins, tannins, alkaloids, amino acids, flavonoids, triterpenes, terpenoids, tannins, saponins, sterols, mucopolysaccharides and other hydroxycinnamic acid derivatives [39,40]. Comfrey contains a wide a variety of chemical constituents, including carbohydrates, tannins, amino acids, allantoin, polysaccharides, triterpenes, sterols, phenolic compounds and pyrrolizidine alkaloids [39,41,42], schematically presented in Figure 1 and summarized in Table 1.

### 3.1. Alkaloids

Many pyrrolizidine alkaloids, known for their high toxicity and biological activity, have been isolated and subjected to a detailed chemical characterization in comfrey [42,68]. Lasiocarpine, echimidine and symphytine have been reported as the most toxic pyrrolizidine alkaloids present in comfrey [45]. Indeed, comfrey leaves have been recognized as having a remarkable health risk, triggering hepatotoxicity in humans and carcinogenesis in rodents, probably attributed to the several hepatotoxic pyrrolizidine alkaloids, including symviridine, symlandine, asperumine, lasiocarpine, symphytine and their related *N*-oxides [40,43,44,49]. As well, 6,7-dihydro-7-hydroxy-1-hydroxymethyl-5H-pyrrolizine, the metabolic product of distinct tumorigenic pyrrolizidine alkaloids in rat liver [69] was linked to its toxic effects [70].

Alkaloids from comfrey have been extensively investigated both quantitatively and qualitatively and identified echimidine, echimidine *N*-oxide, intermedine *N*-oxides, intermedine, 7-acetyl intermedine, 7-acetyl lycopsamine, lycopsamine, lycopsamine *N*-oxides, symphytine *N*-oxide, symphytine, symviridine and symviridine N-oxide as the major constituents. 

In *Symphytum asperum* Lepech, several pyrrolizidine alkaloids were identified in root samples, namely echimidine, lycopsamine *N*-oxides, 7-acetyl lycopsamine, 3-acetyllycopsamine, intermedine N-oxides, symphytine, 7-acetyl symlandine, symviridine, 7-acetyl symviridine, myoscorpine, triangularine and heliosupine [43,44,45]. Similarly, in roots of *Symphytum caucasicum* Bieb., asperumine, echimidine N-oxide, echinatine and lasiocarpine were found as the major alkaloids [44,49]. In the crude extract from roots of *Symphytum cordatum* (L.) W.K. eighteen alkaloids, including echimidine *N*-oxide, 7-sarracinyl-9-viridiflorylretronecine, echimidine, lycopsamine, dihydroechinatine *N*-oxide, dihydroheliospathuline *N*-oxide, lycopsamine *N*-oxide, 7-acetyl lycopsamine-*N*-oxide, symphytine *N*-oxide and 2,3-epoxyechiumine *N*-oxide were tentatively identified for the first time [50]. In *Symphytum tuberosum*, anadoline and echimidine were isolated from the whole plant extract collected in Turkey [66]. Not least interesting in *S. × uplandicum* (syn. *S. peregrinum* Ledeb.), the pyrrolizidine alkaloids, symviridine [43], intermedine, intermedine *N*-oxide, 7-acetyl intermedine, echimidine, echimidine *N*-oxide, lycopsamine, 7-acetyl lycopsamine, acetyl lycopsamine *N*-oxide, symphytine, symphytine *N*-oxide, symlandine, myoscorpine, uplandicine and uplandicine *N*-oxide were determined from leaves and roots [44,57,67]. 

Nonetheless, for *Symphytum officinale* L. were reported, in 1993, that there was no alkaloid-free root in more than 300 samples, coming from over 150 different natural habitats [71]. The content in pyrrolizidine alkaloids (intermedine, acethylintermedine, lycopsamine, echimidine, symviridine) in this plant species ranged from 0.04% to 0.6% (depending on the plant part) [40], being distributed not uniformly, with the concentration in roots being 100-fold higher than the aerial parts [71]. The total pyrrolizidine alkaloid contents ranged from 1380 to 8320 μg/g in root and from 15 to 55 μg/g in leaf [72]. The symphytine and echimidine contents also vary considerably between the tea preparations (comfrey leaves) from distinct suppliers [73], as shown in Table 2 (expressed in ng/g). 

Thus, in *S. officinale* roots twenty three alkaloids were already tentatively identified, including intermedine, lycopsamine, intermedine *N*-oxide, lycopsamine *N*-oxide, 7-acetylintermedine, 7-acetyllycopsamine, 7-acetylintermedinenoxide, 7-acetyllycopsaminenoxide, uplandicine *N*-oxide, myoscorpine, echiumine, symphytine, symviridine, myoscorpine *N*-oxide, echiumine *N*-oxide, symphytine *N*-oxide, symviridine *N*-oxide, heliosupine, asperumine, heliosupine *N*-oxide, asperumine *N*-oxide were identified from *S. officinale* roots [51]. Pyrrolizidine alkaloid lycopsamine, echimidine, echimidine *N*-oxide, symviridine, symlandine, symphytine, symphytine *N*-oxide, intermedine, intermedine *N*-oxide, acetyl intermedine, acetyl lycopsamine, acetyl intermedine *N*-oxide, acetyl lycopsamine *N*-oxide, lasiocarpine, uplandicine and uplandicine *N*-oxide [52,53,54,55,56,57,58]. 

### 3.2. Allantoin

Allantoin was identified in *S. officinale* and *S. cordatum* shoot and root extracts [59]. This molecule was found in roots [39,74] and seeds of *S. officinale* [75]. Allantoin is a metabolic compound of uric acid oxidation products. Natural allantoin is a safe and non-toxic compound, and compatible with cosmetic raw materials for its soothing, skin softening and healing activity [76].

### 3.3. Phenolic Constituents

Trifan et al. reported that comfrey roots are a rich source of phenolic compounds, among them RA and salvianolic acid (A, B and C), widely recognized for its renowned antioxidant effects, contributed positively to the broad spectrum activity of *Symphytum*-derived preparations [41]. The presence of twenty phenolic acids, including caffeic, *p*-coumaric and *m*-hydroxybenzoic acids have been reported in *S. officinale* herb and roots by gas chromatography (GC) and high performance liquid chromatography (HPLC) [65]. RA, *p*-Hydroxybenzoic, caffeic and chlorogenic acids were found in *S. officinale*, and *S. cordatum* shoots and roots extracts [59].

### 3.4. Saccharides

A new anti-complementary dihydroxycinnamate-derived polymer was isolated from *S. asperum* roots [46]. Poly[oxy-1-carboxy-2-(3,4-dihydroxyphenyl) ethylene] was the main component in four water-soluble high-molecular preparations from *S. asperum* and *S. caucasicum* roots and stems. Poly[3-(3,4-dihydroxyphenyl)glyceric acid] was also isolated as a biologically active molecule from *S. asperum* and *S. caucasicum* roots [47]. 

3-*O*-[beta-d-glucopyranosyl-(1->4)-beta-d-glucopyranosyl-(1->4)-alpha-L-arabinopyranosyl]-hederagenin-28-*O*-[alpha-L-rhamnopyranosyl-(1->4)-beta-d-glucopyranosyl-(1->6)-beta-d-glucopyranosyl] ester, a bidesmosidic hederagenin hexasaccharide was isolated from *S. officinale* roots [63]. Both the fructan synthesizing capacity of the different calli and regenerated plants were carefully addressed. Interestingly, the authors found that calli derived from ovaries, anthers and roots were not able to synthesize fructan, while the leaves-derived calli synthesized fructan [62].

### 3.5. Terpenoids, Fatty Acids and Sterols

Symphytoxide A, a triterpenoid saponin was isolated from *S. officinale* roots ethanolic extract [60]. Looking at fatty acid content, the greatest amounts of γ-linolenic acid (16%–72%) were found in a fraction from *S. officinale* seeds [64]. Isobauerenol, β-sitosterol have also been found in *S. officinale* roots [58].

Thus, in general, pyrrolizidine alkaloids were the most often investigated phytochemicals in *S. asperum*, *S. caucasicum*, *S. cordatum*, *S. officinale*, *S. tuberosum* and *S. × uplandicum* roots. Curiously, these biologically active polymers were mostly isolated from *S. asperum* and *S. caucasicum* roots and stems. 

## 4. Traditional Use of *Symphytum* Species 

*Symphytum* species is native to the Western Asia and Europe and has about 35 species [77]. Its folk names include boneset and knitbone. Indeed, the Latin name derives from the Greek *symphis*, which means growing together of bones, and *phyton*, which means plant, broadly referring to its ancient uses. The most common name of comfrey, knitbone, derives from its ability to improve sprains, burning and bruising when used externally. Indeed, *S. officinale* has been widely recommended for more than 2000 years, through internal and external preparations [78], for healing various diseases [79]. Probably, the oldest use of comfrey was topical. For example, comfrey was a remedy for fractures, dislocations and contusions in ancient and medieval times [80]. The farmers used comfrey roots for ointments and tinctures preparation and leaves to treat sprains, contusions, swollen joints or indigestion [81]. *S. officinale* is used in Romania to treat several human and animal disorders [82]. *S. officinale* leaves and roots are externally (i.e., extracts, ointments or compress pastes) used to treat joint disorders, bruises, sprains, pulled muscles and ligaments, hemorrhoids, bone fractures, tendon damage, gastrointestinal (GI) tract ulcerations, lung congestion, joint inflammation and in wound healing promotion [3,78], inflammatory disorders of wounds, joints, distortions, hematomas and thrombophlebitis [5]. As infusions and extracts, these plants are used for internal applications in treating gastritis, gastroduodenal ulcers, and lung congestion [3,77,83]. In the German system of medicine, *S. officinale* has been used against skeletal muscle disease [77,80] while in Brazil, its infusion is used for GI problems (i.e., liver disturbances, gastritis and ulcers) [84]. In Mexico and Turkey aerial parts are used for the native treatment of rheumatism [85] and gastrointestinal ulcers, respectively [77]. In USA, it is used for skin problems [86]. In Lithuania, roots are used through the form of teas or ointments for bone pain and contusions [87]. *S. officinale* and *S. tuberosum* root based clay balms are externally applied for bone breakages, sprains and rheumatism was reported in Navarra [88,89].

## 5. Biological Activity of Comfrey

There has been growing importance in searching safer and effective food preservatives and antimicrobial agents primarily derived from natural matrices towards finding new chemical structures for counteracting food spoilage and microbial resistance [90]. The widespread use of antibiotics in agriculture, medicine and veterinary has markedly triggered the development of increasingly resistant microbial strains, hampering the eradication of pathogenic microbes [91,92,93,94] consequently. There has been an increasing knowledge of how distinct plants and its derived extracts favor wound healing, including the ability to act as antimicrobials, antifungals, astringents and so on. Koll et al. attributed the therapeutic properties of *S. officinale*, mostly to the anti-inflammatory, analgesic and anti-exudative effects, as also to its ability to stimulate granulation and tissue regeneration [95].

### 5.1. Potential Application as Food Preservatives

Food preservatives are often used with the key purpose of preserving the foods’ natural characteristics and appearance, and to increase its shelf value during storage. Thus, food preservation is of great interest as it resides in the role that foodborne disease plays in human health and well-being [96]. In 2010, the World Health Organization (WHO) stated that thirty-one global hazards (e.g., virus, bacteria, protozoa, helminths and chemicals) were responsible for causing 600 million foodborne illnesses and 420,000 deaths [97].

Nowadays, conventional preservatives are synthetic chemical substances, which include some that are being re-evaluated, e.g., nitrates and nitrites by the corresponding authority due to their potential health risk [98]. In fact, over 60% of the population recognized to worry about the presence of food additives, including food preservatives in foods in 2010 [99]. For that reason, new natural compounds are being investigated to be used as food preservatives. This new trend is called biopreservation, which broadly conceives the application of plants, animals, microorganisms and/or their metabolites in the enhancement of food shelf life and food safety [100,101]. In fact, traditionally, many plant-derived preservatives, e.g., vinegar, citrus fruit juices and some plant extracts have been employed to preserve foods for centuries [102].

The access to these natural food preservatives increases in importance in developing countries where the resources to preserve foods is scarce. Their protective effect has been associated with their composition in bioactive compounds such as phytochemicals, which protect them against viruses, bacteria or fungi. Despite *Symphytum* species, and especially *S. officinalis*, have been commonly employed as a topical anti-inflammatory [4], there is a scarcity of information regarding the use of comfrey for food preservation. However, due to its chemical composition, which includes allantoin, polysaccharides, glycopeptides, amino acids, triterpene saponins, alkaloids or phenolic compounds, among others, they should not be discarded as potential food preservatives. The most known preservatives in foods are antimicrobial and antioxidants agents. In this regard, Table 3 shows the antimicrobial, antifungal and antioxidant activities of comfrey.

Food antioxidants are mainly employed to protect food from oxidants, which can alter their organoleptic, texture and safety properties [103]. For that purpose, the European Food Safety Authority (EFSA) has approved several food antioxidants including ascorbates, tocopherols, gallates, erythrobates, butylates, lactates or rosemary [103]. Among the *Symphytum* species, one of the most studied is *S. officinale*. Specifically, *S. officinale* roots have been demonstrated to be a good source of phenolic compounds [74]. Sowa et al. also found five phenolic acids in an *S. officinale* root extract, including RA, which showed a high in vitro antioxidant activity [9]. In the agreement, Trifan et al. quantified several phenolic compounds in a comfrey root extracts including RA as the major one followed by salvianolic acids isomers. RA has been highlighted as the most abundant with about 8 mg g^-1^ extract [41]. In the same work, they showed higher antioxidant activity of a comfrey root extract compared to standard antioxidants through different methods, thus, evidencing that phenolic compounds seem to display a crucial role in comfrey root extract antioxidant effects [41]. Similarly, Neagu et al. described the higher antioxidant activity of comfrey extracts processed by ultrafiltration than ascorbic acid [39], a common additive already used as a food preservative (E-300) [98].

Within the food preservatives employed as antimicrobials are found sodium benzoate, calcium propionate and potassium sorbate, among others [102,104]. On the other hand, the most recognized natural plant-derived antimicrobial compounds encompass phenolic compounds, essential oils or antimicrobial peptides [100,105]. Nevertheless, alkaloids, naturally occurring organic compounds in plants, have shown to exert broad-spectrum antimicrobial effects [106,107,108]. In this regard, alkaloids represent another group of important compounds described in *Symphytum* species [109]. Interestingly, they found higher concentrations in comfrey leaves than in other plant parts, and are precisely the leaves that have been traditionally consumed as a tea [73]. However, several studies showed that pyrrolizidine alkaloids could be hepatotoxic [110,111]. They are not exclusively present in *Symphytum* species since they can be found in more than 300 plant species [40,110].

Interestingly, earlier studies have demonstrated that alkaloids isolated from *Symphytum sylvaticum* leaves and roots are effective against several fungal cultures in vitro, with the root alkaloid extract rich in echimidine-*N*-oxide being more protective against certain fungi than leaves [112]. Early studies also carried out with comfrey leaves with a high content of phenolic compounds calculated per unit of biomass than the leaves demonstrated to be effective against the germination of conidia and uredospore of pathogenic fungi when the aqueous extract was spraying wheat seedlings [20]. On the other hand, Savić et al. did not find any activity of a comfrey root aqueous extract against *Aspergillus niger* and *Candida albicans* [113].

*Symphytum* species’ antibacterial effect is scarcely explored so far. Sumathi et al. investigated the potential of several comfreys leaves extracts against several pathogenic strains, i.e., *Staphylococcus aureus, Bacillus subtilis, Pseudomonas aeruginosa, Salmonella typhi, Escherichia coli* and *Proteus vulgaris*. They found that comfrey leaves extract exhibited partial and strong inhibition against *S. aureus, B. subtilis, P. aeruginosa* and *S. typhi* [114]. Though authors did not attribute its effect to a specific group of compound, the antimicrobial mechanism of action of comfrey leaves might be due to its composition in phenolic compounds, which could act by interfering with bacterial cell permeability, binding to adhesins or inhibiting the DNA or RNA replication and transcription [100]. Whilst comfrey leaves also showed an inhibitory effect against *Bacillus cereus*; their roots extract presented a maximum inhibitory effect against *Proteus vulgaris* and *S. aureus* [115]. *E. coli* and *Salmonella typhimurium* were also highly sensitive to comfrey roots aqueous extract [113]. Contrarily to the results mentioned above, Woods-Panzaru et al. did not find any antimicrobial activity against 34 microorganisms, including 24 bacteria and 10 fungi of 50 mg/mL of comfrey leaf extract [116]. Thus, the lack of conclusive and strictly focused studies on food microbial and fungal trigger the need for increasingly detailed researches to assess the real antifungal and antibacterial effects of comfrey roots and leaves. Despite the lack of comprehensive compositional studies, preliminary data suggests that this natural matrix contains a wide pool of bioactives for further use as food preservatives (Table 3). Nevertheless, more studies are necessary to elucidate the full chemical composition of different *Symphytum* species, their bioactivity as well as their toxicity.

### 5.2. Antimicrobial Activities of Symphytum Species

Traditional medicine has occupied a prestigious place in the search for effective strategies to treat various diseases, given the high rates of microorganisms with acquired resistance to the currently available antibiotics, as well as to discover new substances [118,119,120,121]. In several countries, it is common to use plants and their parts in disease treatment, and WHO (2014) reported the important role of traditional medicine highlighting different forms and specific types of this medicine [122]. Comfrey is well recognized as useful for medicinal preparations, due to their healing and therapeutic properties. The present section assessed the possible antimicrobial activities of these plants by pre-clinical studies. Table 4 summarizes the evidence regarding the in vitro antimicrobial activities of *Symphytum* species. The reported studies present the antimicrobial activities by measuring zone diameters of growth inhibition. Different solvents were used to obtain the corresponding extracts from the distinct plant parts. Overall, results indicated that comfrey extracts have potential antimicrobial effects on several bacterial strains tested, especially *S. aureus* as well as antifungal activity against *Bipolaris oryzae* [123]. Rocha et al. suggested a possible biological control of the endophytic strains from *S. officinale* leaves against *S. sclerotiorum* [124]. Moreover, Karavaev et al. stated that comfrey leaves extracts decreased the susceptibility to infection from *Erysiphe graminis* conidia and *Puccinia graminis* uredospores in wheat steam [20]. It should be highlighted that the effect of the investigated extracts results from a mix of compounds, including phenolic compounds (caffeic and chlorogenic acids, allantoin and luteolin glycoside), possibly due to the synergy between compounds, as well as with other bioactive molecules present in the whole plant.

Some time ago, Dolganiuc et al. demonstrated an alternative modality to antimicrobial inhibition using *S. officinale* roots aqueous extract in mouse peritoneal macrophages through the activation of the cell respiratory burst and inhibition of catalase and superoxide dismutase (SOD) synthesis, among others [130]. 

As aforementioned above and considering the great potential of some secondary metabolites to inhibit the growth of certain pathogens, the reviewed pre-clinical studies regarding comfrey antimicrobial activities allows us to summarize the following key points: i) Investigations on multiple bacteria and fungi to assess the spectrum activity of such plant parts extracts are needed; ii) single or pooled parts of the plants are to be assessed for their antibacterial activity at increasing doses and using different aqueous/organic solvent extracts; iii) isolation and chemical structure determination of bioactive compounds could improve the clinical intervention in infections treatment triggered by pathogenic multidrug-resistant bacteria and iv) the in vivo antimicrobial effect of comfrey alone should be demonstrated.

### 5.3. Antioxidant Activities of Symphytum Species

A wide variety of reactive oxygen species (ROS) has been related as being in the genesis of human diseases (e.g., inflammation, autoimmune deceases, neurodegenerative disorders, viral infections, digestive system, among others), and are currently the focus of multiple studies and led to numerous advances [90,131]. Various studies have reported that some scavengers react with ROS, through a direct or indirect manner, and defuse their reactivity to bounds oxidative damage [132]. Potential of natural compounds as antioxidant agents are the topic of current interest that is being studied by antioxidant properties in vitro and in biological systems [131]. Several natural antioxidants derived from plant sources have shown to act as active oxygen scavengers, free radical inhibitors or reducing agents [133]. Specifically, comfrey-derived products are largely applied in traditional medicine for multiple pharmacological purposes. As they promote the immunological status, wound-healing, antioxidant effect and anti-inflammatory that oxidation mechanisms and free radicals can play a role in these process [42]. Several literatures have reported antioxidant activity of various *Symphytum* species. Different plant parts including root, stem and leaves have investigated for antioxidant efficacy, and some are listed in Table 5.

Most of the studies have been done on *S. officinale* root and its components, namely on RA as a natural polyphenol antioxidant; allantoin as an anti-inflammatory and autoimmune enhancer agent and tannins as anti-inflammatory agents [41,138], and correlated with oxidative stress [39]. Indeed, different studies demonstrated the noteworthy antioxidant activities of comfrey extracts compared to known antioxidants [8].

It has been proved that *S. officinale* polar extracts have higher amounts of polyphenols, and demonstrated higher antioxidant effects. The comparison of comfrey root ethanolic and aqueous extracts have demonstrated a higher radical scavenging activity to the ethanolic extract (as strong as ascorbic acid), composed of higher contents of phenolic compounds than the aqueous extract [77]. Similarly, Sowa et al. found a significant antioxidant effect of ethanol/water extract of comfrey root, which was linked to its high total phenolic content and high phenolic acids concentration, including RA, *p*-hydroxybenzoic, caffeic, chlorogenic and *p*-coumaric acids, but especially RA. While water extract had fewer phenolic acid and less antioxidant activity [9]. The same composition of phenolic acids have already been reported for concentrated root extract that showed increasing of active principle by micro and ultrafiltration of ethanolic extract, rose the DPPH inhibitory effect even higher than Trolox inhibition activity [134]. The association between phenolic compounds and radical scavenging effects was also confirmed by Neagu et al. via ABTS and DPPH methods. As concentrated extracts of *S. officinale* root were obtained through ultrafiltration contained more biologic active principles and showed high antioxidant activity (above 90% DPPH inhibition) [8]. Comfrey root has a rich pool of phenolic compounds with remarkable antioxidant effects. RA and salvianolic acid comprise the most often present, having the ability to scavenge free radicals and chelating metal ions as efficient antioxidants [41]. Polar extracts of *S. officinale* leaves also have revealed an antioxidant potential. Study on methanol/water, ethyl acetate and water extract proved methanol extract exhibited the highest polyphenols concentration and most power radical scavenging activity by ABTS and DPPH methods [136]. Antioxidant evaluation of the leaves extract in another study showed that DPPH and superoxide radical scavenging activity of the ethanolic extract is more than the aqueous extract, and was correlated with total phenolics content [77]. Some other researches show that the antioxidant activity of comfrey is associated with the phenolic polymers. Recently, naturally-occurring polysaccharides have gained pivotal attention among scientists due to their antioxidant and scavenger activities [139]. Several studies on scavenger activities of purified plants polysaccharides, crude polysaccharide extracts from different medicinal plants proved their noteworthy antioxidant activities as free radicals’ scavenger, antioxidant enzymes enhancer, lipid oxidation inhibitor and advocated that plant carbohydrates and water-soluble polysaccharide complex fractions can be probed as novel antioxidants [139,140]. Their antioxidant activity is depended on the chemical and monosaccharide composition and molecular weight, as the mentioned items affected their electron-donating ability. Duan et al. already reported that different extraction techniques lead to several differences in polysaccharides (Ps) characteristics, yield and antioxidative potential isolated from *S. officinale* aerial parts. Maximum extraction yield, smallest molecular weight, upper uronic acid content and best antioxidant capacity were found in Ps extracted by ultrasonic-assisted technique versus hot water extraction Ps that showed the lowest yield and antioxidant activity [141].

Strong inhibition of DPPH radical and of non-enzymatic lipid peroxidation capacity in bovine brain extracts were reported to a water-soluble hydroxycinnamate-derived polymer from *S. asperum*, more prominent than that of quercetin, butylated hydroxytoluene (BHT), and ascorbic acid. The anti-lipoperoxidative evaluation showed a dose-dependent effect, where 10 ng of *S. asperum* polymer inhibited 50% of lipid damage, while the IC_50_ of quercetin as reference material is 2 µg/mL. It can be concluded that this polymer has an anti-peroxidant activity about 200 times higher than that of the standard [132].

Among the two high-molecular-weight water-soluble biopolymers, the major one was poly[3-(3,4-dihydroxyphenyl) glyceric acid] or poly[oxy-1-carboxy-2-(3,4-dihydroxyphenyl) ethylene] isolated from *S. officinale* roots, and exhibited antioxidant effects by interfering in active oxygen species (AOS) formation, by polymorphonuclear neutrophils (PMN) and AOS binding directly without PMNs cytotoxic effects [117]. The effect of phenolic polymer fractions from *S. asperum* and *S. caucasicum* roots and stems [48] and leaves [10] were studied by luminal and lucigenin induced chemiluminescence (CLlum and CLluc, respectively) test to evaluate their inhibition ability on ROS produced by human polymorphonuclear neutrophils (PMNs; activated by opsonized zymosan (OPZ) and phorbol-myristate acetate (PMA)) and also on CLluc in cell-free hypoxanthine–xanthine oxidase (HX/XO) system to assay superoxide anions scavenging ability. The results exhibited a noticeable antioxidant activity for all fractions, where the major one was poly[3-3,4-dihydroxyphenyl) glyceric acid] in roots and stem extracts [48], already reported in the root of both *Symphytum* species [142], although this polymer was not identified in the extract from leaves [10].

In summary, comfrey has significant antioxidant effects, due to their bioactive compounds, and therefore, they can be considered as a factor affecting the healing process. Their pharmacologic effects, like anti-inflammatory and wound healing can also be justified by its antioxidant activity mechanisms.

### 5.4. Hepatoprotective Effect

*S. officinale* ("black root" or in Italian "radice near") is a popular medicine in Moldova, commonly used for chronic hepatitis and hepatocirrhosis. The alcoholic extract of its roots was tested in vivo on 42 albino rats (180–240 g), orally administered at 0.4 g/kg/day during two weeks [143]. The chronic toxic hepatitis was induced by injection of 20 mL/kg tetrachloromethane twice a week for two months. The results obtained were compared to the standard drug Silibor (30 mg/kg/day) for two weeks. As for the main findings, *S. officinale* extract displayed similar or higher effects than Silibor, acting through decreasing lipodystrophy and limiting the conjunctive proliferation in the interlobular grooves.

### 5.5. Effect on Musculoskeletal Disorders

Oberbaum et al. induced bone fractures in guinea pigs and had shown that *S. officinale* could promote the healing of fractured bones. Indeed, after X-ray and histology analysis, the authors stated that the animals receiving either of the two *S. officinale* dilutions and irrespective of the mode of administration presented their new bones completely mineralized through the entire fracture site in 33% of the fractures [144].

Araujo et al. reported that an emulsion containing 8% comfrey leaves extract triggered damaged tissue repair, improved collagen deposition and reduced cellular inflammatory infiltrate by 46% in an open wound in a rat model [145].

### 5.6. Wound Healing Activity

Alkan et al. showed that *S. officinale* extracts have proliferative activity on 3T3 Swiss albino mouse fibroblast cells using MTT (3-[4,5-dimethylthiazol-2-yl]-2,5-diphenyl tetrazolium bromide) and neutral red uptake (NRU) assays, which seems to be beneficial for wound healing [77]. The Caucasian comfrey species, *S. asperum* and *S. caucasicum* have been mainly recommended in traditional medicine for fractures and wounds treatment, as they contain allantoin. Indeed, as previously reported, allantoin promotes cell division and connective tissues, bones and cartilages growth and, thus, it is responsible for wounds healing [146]. The extracts also contain hepatotoxic pyrrolizidine alkaloids, which markedly conditionate the internal use of comfrey. Barbakadze et al. assessed the in vitro (antioxidant and anti-complementary) and in vivo (rat models with wound and skin burn) pharmacological effects of allantoin and toxic pyrrolizidine alkaloids-free composition containing crude polysaccharides and a novel biopolymer from *S. asperum* roots—poly[3-(3,4-dihydroxyphenyl) glyceric acid] (PDGA), and found that PDGA exhibited a prominent antioxidant and anticomplementary activity, while polysaccharides displayed no detectable anti-complementary and antioxidant potential. An ointment preparation containing 2.5% crude polysaccharides and PDGA displayed prominent wound healing effects, by efficacy not yielding to 2.5% allantoin ointment. Thus, given the high degree of reliability observed for comfrey preparations, it is feasible to suppose that it may be used both with allantoin and PDGA [117]. Savić et al. assessed the in vitro bioactive effects of pure allantoin and comfrey roots-derived aqueous extract, standardized to the allantoin content. Pure allantoin (40 and 100 μg/ml) displayed mild inhibitory effects on epithelial (MDCK) and fibroblastic (L929) cell lines proliferation using an MTT test, although more prominent on MDCK cells. On the other hand, comfrey root-derived aqueous extract (>40 μg/ml) exerted pronounced stimulatory and inhibitory effects on L929 and MDCK cells proliferation, respectively. Thus, it is possible to infer that the botanical preparation derived from the aqueous extract has higher anti-irritant potential than pure allantoin. Furthermore, it was observed that creams exert a higher effect on hydration and erythema index (EI) compared with gels containing the same constituents. These findings indicated that comfrey root extracts bioactive effects are not only attributed to allantoin, but also other chemical constituents. Thus, comfrey extract containing topical preparations may confer a plus in the treatment of skin irritation [113].

### 5.7. Anti-Inflammatory and Antinociceptive Activity

Vostinaru et al. assessed the anti-inflammatory and antinociceptive effects of an *S. officinale* root extract, containing 74.77 μg/mL RA. Carrageenan-induced rat paw edema was used to assess the hydro-glyceroalcoholic extract (500 mg/kg, orally) anti-inflammatory effect. This extract revealed to be able to trigger a 55.6% reduction in edema at 1 h after inflammation was induced. The antinociceptive effect, the extract caused 45.25% inhibition of acetic acid-induced abdominal constrictions in mice, and in Randall Selitto test in rats, the same dose administered orally led to a strong peripheral antinociceptive effect during the first 2 h post-administration, increasing the threshold pain by 58% [147]. A glycopeptide isolated from *S. officinale* roots aqueous extract also induced an antichloristic effect on carrageenan-induced rat paw edema in a dose-dependent manner, inhibiting prostaglandins and leukotrienes release, through decreasing phospholipase A2 expression [148]. Indeed, it has been stated that *S. officinale* anti-inflammatory activity is mostly attributed to its content in triterpenes, which inhibit different stages of inflammation (e.g., histamine release, cyclooxygenase (COX) and lipoxygenase (LOX) activity and nitric oxide production), and polyphenols responsible for triggering a selective COX-2 inhibition [149]. 

## 6. Clinical Effectiveness of *Symphytum* Species in Humans

According to the European Scientific Cooperative on Phytotherapy Monograph (ESCOP) monograph, comfrey root is mostly recommended to be used for tendinitis, knee joint injuries, gonarthrosis, fractures and skin inflammation (ESCOP 2009). *Symphytum* species (most commonly *S. officinale* and *S. × uplandicum*) preparations have strong clinical records, which substantiate their longstanding traditional topical use in the treatment of musculoskeletal problems such as osteoarthritis (OA), back pain, ankle sprains, joint distortion, myalgia and rheumatism. Clinical effectiveness of the topical preparations containing either a pure extract from comfrey roots (known as *Symphyti* radix) or their combinations with marketed formulas has been well established through individual case reports, clinical trials and post-marketing surveillance (Table 6).

### 6.1. Wound Healing

#### 6.1.1. Chronic Marginal Parodontopathies

Gafar et al. reported the antimicrobial, antimycotic and anti-inflammatory effects of propolis and *S. officinale* extracts-based original products in chronic marginal parodontopathies treatment [164]. 

#### 6.1.2. Blunt Injuries

Blunt injuries are amongst the most common areas for topical application of comfrey, given its renowned anti-inflammatory and analgesic effects, and ability to stimulate granulation and tissue regeneration. The percutaneous efficacy of the aforementioned commercial product (Kytta-Salbe® f), composed of 35% liquid comfrey root extract was evaluated in the randomized, placebo-controlled trial on 142 patients with unilateral acute ankle sprains, over eight days [95]. Patients received four treatments/day. Comfrey-treated patients evidenced a significant amelioration with regards to the pain and ankle edema reduction, ankle mobility and global efficacy when compared with control patients. Under active treatment, there were no drug-related side effects. In general, this trial confirmed the promising analgesic, anti-inflammatory and anti-exudative effects, and good tolerance of the comfrey product.

#### 6.1.3. Acute Blunt Traumata

Fresh abrasions: A study design (randomized, double-blind clinical trial, verum vs. reference) was used to assess wound healing effects of the preparation (Traumaplant^®^) in 278 patients with fresh abrasions [159]. A faster and clinically relevant initial reduction in wound size (49% ± 19% vs. 29% ± 13%) was obtained in favor of verum, 2–3 days post-application. A good to very good efficacy was rated by physicians in 93.4% of cases in the verum group when compared to 61.7% of cases in the reference product-treated group. In the case of drug-related adverse reactions, none of the patients experienced problems for the 10 day observation period. Barna et al. further confirmed the wound healing potential of Traumaplant^®^ preparation, topically applied, composed of 10% active ingredient isolated from *S. × uplandicum* Nyman aerial parts [161]. Data for evaluating the overall Traumaplant^®^ efficacy and tolerability have also been available in children. Another study assessed the therapeutic applicability and safety of the product in 196 children (4–12 years) for acute blunt traumata treatment. A clear improvement, ranging from 84.5% to 100% was found for every individual parameter, such as pain on palpitation, pain in motion, functional impairment, edema and hematoma. Concomitantly, excellent tolerability and compliance were observed [160]. Later, the data from this non-interventional study were confirmed and extended with a randomized, controlled, double-blind clinical trial, composed of 108 children, from three to 12 years (*n* = 54/group) with fresh abrasions [161]. The design of the study included the comparison of the 10% extract (Traumaplant^®^) with a low dosed 1% reference. A healing rate of 50% was stated 0.9 days earlier in the high concentration-treated group when compared to the lower concentration group. Physicians and children/parents classified the efficacy of the 10% cream as better than that of the control preparation (1% formulation). No drug-related adverse effects or tolerability-related problems were reported. The findings of both observational and clinical studies in pediatric patients mentioned above demonstrated the effective and safe use of topical comfrey extract isolated from *Symphytum* aerial parts in a blunt traumata treatment. However, given safety concerns controlled/multi-center clinical trials are needed on the use of comfrey extracts in children.

#### 6.1.4. Venous Ulcers

More recently, Oreščanin reported an achievement within two months under application of a topical treatment with Bioapifit^®^, a mix herbal wound healing ointment, in elderly with venous ulcers. The ointment applied resulted in strong wound healing, hemostatic, anti-inflammatory and antimicrobial potential [165]. The same author even suggested the alternative use of this herbal preparation, instead of topical corticosteroids and immunomodulant therapy, in the treatment of mild-to-severe atopic dermatitis in infants/children. The ointment mentioned above resulted in complete and/or strict remission of symptomatology [165]. Oreščanin attributed the observed effect to the ointment formulation, which contains in its composition substances with prominent emollient, anti-inflammatory, immunomodulatory, wound healing and antimicrobial potentialities, that simultaneously targeted various mechanisms involved in atopic dermatitis pathogenesis, in children [165]. In a pilot study, Binic´ et al. evaluated the healing and antimicrobial potential of herbal therapy on a venous leg ulcer. The topical treatment with Plantoderm^®^ ointment counteracted the polymicrobial effects of *S aureus*, *P. aeruginosa*, *E coli* and *P mirabilis* in a non-infected venous leg ulcer [166].

#### 6.1.5. Squamous Endocervical Metaplasia

Oreščanin et al. tested the efficiency of newly developed herbal pessaries for the treatment of the squamous endocervical metaplasia in women aged 20–59 years [167]. The wound healing, anti-inflammatory, antimicrobial and antiviral effects of the pessaries could be due to the synergistic effect of pooled medicinal plants. In a more recent conference paper (2017), it was revealed that BIOAPIGYN^®^ ointment application was effective in treating lower genital tract infections, involving *U. urealyticum* and *E. coli*, while no effects were stated against *M. hominis* and *Candida* species. The achievement goal was probably due to the significant pro and prebiotic effects and low pH of the ointment constituents [167].

#### 6.1.6. Skin Disorders

Comfrey plant-derived extract extracts have also been increasingly used for treating some skin diseases, including erythema, chronic varicose ulceration and decubital ulcers. Although there is a considerable amount of clinical and observational data assessing its effects in musculoskeletal diseases, the application of comfrey extracts in skin disorders is limited with experimental studies and individual case reports. In 29 volunteers, topical preparations with 5% or 10% comfrey root extract (2:7, 50% ethanol) were assessed for its healing effects in experimentally induced UV-B erythema [162]. The studied extracts revealed an anti-inflammatory potential similar or greater than that of diclofenac. Recently, the efficacy of 10% topical comfrey extract preparation (Traumaplant^®^) was evaluated in an open, prospective study with the involvement of 161 patients who were in the second and third stage of decubitus ulcers (pressure ulcers, intention-to-treat (ITT) population). The bandages with the cream were used for the application, being changed every 2–3 days during four weeks. The main parameters of efficacy included the sore area and wound depth (planimetrically in mm). Accordingly, in the case of the total decubitus area 89.2% reduction was observed and a similar result (88% reduction) was also obtained for pressure ulcer depth. Only two cases of local irritation were stated post-treatment period. Therefore, this study confirmed that comfrey extract is highly effective in pressure ulcers treatment and also it shows excellent skin compatibility in the case of open sores [163]. However, taking into consideration the general rule, more human studies are needed to justify both the efficacy and safety of comfrey extracts in treating skin problems.

### 6.2. Musculoskeletal Problems

#### 6.2.1. Osteoarthritis

Several clinical trials were carried out to assess the effect of different comfrey root preparations in osteoarthritis (OA)-related symptoms management. According to the World Health Organization, half of the worldwide population aged 65 years and over has OA, which is the most prevalent disorder of articulating joints in human and is restricting the quality of life. Therefore, pain reduction, joint’s function preservation and restoration are important therapeutic objectives [168]. Other researchers studied the effect of comfrey root extract-based cream (10% or 20%), containing tannic acid and eucalyptus oil, when compared to a placebo cream and a standard cream, containing only eucalyptus in 43 subjects (45–83 years old), diagnosed with primary knee osteoarthritis. Participants used the extract-based cream for six weeks, three times daily and were assessed every two weeks over the treatment. Significant differences were stated in osteoarthritis index categories at *p* < 0.01 (pain, stiffness and physical functioning), confirming that the two concentrations of comfrey-based creams used were higher to that of the *Eucalyptus* reference cream. In each group, two participants evidenced temporary and minor adverse reactions, manifested as skin rash/itching, which were rapidly solved through modifying its use (Smith and Jacobson 2011). The earliest study was performed in 61 OA knee patients by using a cream formulation containing comfrey root extract (unknown species) and mistletoe. A significantly greater reduction was observed in morning/evening pain (both 28%) and night pain (51%) in the experimental group, receiving 1.7 g herbal preparation when compared to the placebo [150]. Another clinical trial was conducted over 21 days in 220 painful knee OA patients [151]. All patients received either a commercial preparation (Kytta-Salbe^®^) or a placebo cream three times/day. After the study period, pain in the verum group was reduced five times (reduced from moderate to mild) more than in the placebo group. The first target variable was VAS (visual analog scale: Total score of pain at rest and pain on movement) improved 54.7% in the verum group and only 10.7% in the placebo group. Regarding the second target variable— Western Ontario and McMaster Universities Arthritis Index (WOMAC) total score, which measures pain, stiffness and functional limitation, the verum group proved to be four times more effective than placebo. Overall, this study revealed to be well conducted, following the Good Clinical Practice-International Conference oh Harmonization (GCP-ICH) guidelines, and with regards to these findings, the short-term use of this product may be an effective treatment strategy for painful OA knee. Another randomized controlled, multicenter clinical trial conducted on 43 patients with OA knee included the application of the marketed comfrey-based botanical cream 4Jointz, composed of a blend of tannic acid and eucalyptus, three times/day for six weeks [138]. Two comfrey root extract-derived ointments, at distinct concentrations (10% and 20% strength by volume) were compared with a pseudo-placebo of eucalyptus, similar in color, texture and smell to the experimental ointments. Results were determined every two weeks during the treatment period. Both formulas were interesting in alleviating pain and stiffness and in improving daily function within the first two weeks of treatment. This amelioration was maintained every two-week assessment period, up to the end of the study, being both 10% and 20% comfrey-based creams higher in terms of efficacy than the eucalyptus reference cream. Two subjects evidenced temporary and minor adverse reactions, manifested as skin rash/itching, but were rapidly solved through modifying its application. In another placebo-controlled trial, a 4Jointz cream combination was assessed in 133 OA patients [152]. Several parameters were evaluated, such as pain, inflammation markers and cartilage breakdown, over 12 weeks. At the end of the study, pain scores were significantly diminished in the 4Jointz group when compared to the placebo. Scores were assessed using the VAS pain intensity and the Knee injury and Osteoarthritis Outcome Score (KOOS) pain scales. No significant changes were stated to IL-6 and CTX-2. Therefore, it can be inferred that 4Jointz topical treatment was able to reduce pain, although no effects were observed on inflammation or cartilage breakdown over the 12 weeks treatment. Overall, the findings of all these randomized, controlled clinical trials strengthen the efficient comfrey root extract used in the symptomatic relief of OA and also establish its place as an alternative option to other topical preparations [152].

#### 6.2.2. Back Pain

Formulations containing comfrey root extracts have also been considered an effective alternative for back pain treatment, a very common health problem affecting physical performance and general well being worldwide. A clinical trial was conducted in 120 patients with acute upper and lower back pain over five days [153]. The patients were administered with 4 g of the licensed medicinal product (Kytta-Salbe^®^ f-mentioned above) containing comfrey fluid root extract or placebo per application, three times a day. The trial included four visits. A pronounced difference was stated between comfrey root extract and placebo groups. During the trial, pain intensity on active standardized movement decreased around 95.2% and 37.8% in the verum and placebo groups, respectively. With this study, a quick-acting effect of the comfrey root extract was also formerly addressed. In the comfrey group, the pain intensity was reduced by about 33% after 1 h. However, the rate of the decrease was just 12% in the placebo group. Comfrey root extract displayed a prominent ability to reduce acute back pain, being also free of serious adverse reactions. The combination of the same comfrey root extract cream with 1.2% methyl nicotinate (Kytta-Balsam Wf) was also investigated in a randomized, multicenter, three-arm, placebo-controlled trial [154]. The ability of this combination to relieve acute upper or lower back pain was compared with a single methyl nicotinate preparation or with a placebo cream in 379 patients who were administered by a cream three times/day over five days. It was demonstrated that patients treated with the topical combination of comfrey root extract and methyl nicotinate presented a more pronounced reduction in pain scores and increased tenderness. As a result, it was stated that methyl nicotinate contributed verily to product efficacy, even reducing the primary efficacy parameter by 31% when compared to the placebo group. The 22 drug-related adverse effects, which were classified as mild to moderate, occurred in combination and methyl nicotinate groups; however, none in the placebo group. Pancreatic insufficiency and recurrent depression were two series of adverse effects observed in the combination treatment group.

#### 6.2.3. Epicondylitis, Tendovaginitis and Periarthritis 

In one of the earliest randomized pilot studies, a comfrey root ointment (*n* = 20) or a placebo ointment (*n* = 21) were administered for four weeks in patients suffering from several kinds of musculoskeletal rheumatism [155]. Patients pain was investigated using distinct clinical parameters, such as pressure’ tenderness, pain at rest and on exercise. After two weeks, patients with epicondylitis and tendovaginitis and treated with the comfrey ointment presented a clearly better efficacy when compared to the placebo with regards to pressure’s tenderness. However, periarthritis-treated patients did not evidence any positive effect in relation to the placebo, even after four weeks.

#### 6.2.4. Acute Ankle Distortion

Predel et al. performed a clinical trial, aiming to assess the effect of a commercial product (Kytta-Salbe^®^-f), containing 35% liquid comfrey root extract (*n* = 82), or Diclofenac gel (*n* = 82), four times/day in patients with acute unilateral ankle sprains, over one week [156]. The authors strive to investigate both the efficacy and tolerability of percutaneous comfrey ointment application with that of a diclofenac gel, which crosses the skin barrier, reaching joints, muscles and synovial fluid and exerts locally a highly potent effect [169,170,171]. In this study, the former variable of efficacy was determined as pressure-derived pain in the injured area. Secondary variables were defined as a joint circumference and individual pain sensation while resting and in movement. Considering both primary and secondary variables, comfrey extract displayed a more prominent effect than the diclofenac. After seven days of treatment, the ankle swelling decreased by 79.5% in the group treated with the comfrey root extract, while the decrease in the diclofenac group was 69.4%. Pain on pressure, measured by an algometer, also was reduced by 80.6% and 74.7% by the comfrey root extract and diclofenac group, respectively. By considering several different parameters, the re-evaluation of the trial according to the Committee for Proprietary Medicinal Products (CPMP) guidelines also exhibited the superiority of the comfrey preparation over the diclofenac gel [172]. Another clinical trial investigated both efficacy and safety of Traumaplant ^®^ (containing 10% comfrey extract) in 203 patients with acute ankle distortion when compared with a reference product containing a 1% active ingredient [157]. Potentially toxic PAs were not present in the preparations for both verum and reference groups (detection limit <0.1 μg/g). In this study, reduction of pain on active motion and at rest, and a functional compromise was found to be more significant and clinically relevant at the 3rd, 4th and 7th day, using the highly concentrated product (*p* < 0.001). Swelling amelioration was also more significant on day 3–4, when compared to the reference product (*p* < 0.01). Accordingly, the overall efficacy was determined as good to excellent in 85.6% and 65.7% of verum and reference cases, respectively, at the 3rd and 4th day. Tolerability was found to be excellent. Overall, this study confirmed the positive benefit-risk ratio and underlined that a moderately high concentration of extract is needed to reach a great effect. In one such open, uncontrolled study, the effects of a comfrey aerial parts extract were investigated. The experimental ointment was applied in the locomotor system of 105 patients with painful disorders, twice a day. Very interesting results were stated in subacute and chronic complaints treatment, accompanied by functional symptoms reduction, like swellings (90%–94 % of patients). In half of the patients (57 out of 105), functional disturbances and pain were also completely resolved [80].

#### 6.2.5. Myalgia

The above-mentioned topical product, Traumaplant^®^ with 10% active ingredient, was assessed and compared for its effectiveness and tolerability in 215 patients with myalgia, with a reference product, with a 1% active ingredient [158]. Pain in motion was defined as the primary efficacy parameter, and pain at rest, pain on palpation and functional impairment as secondary efficacy parameters. Using Traumaplant^®^ at a 10% active ingredient, pain on active motion, at rest and on palpation were significantly reduced when compared to the reference product. It was clinically evident that the high concentration of *Symphytum* active ingredients present in Traumaplant^®^ was closely related to the rapid decrease in pain when compared with the reference product. With regards to overall tolerability, it was classified as excellent, as no systemic adverse effects were stated.

Data from all these randomized, controlled clinical trials established the efficient use of comfrey root extract in the treatment of musculoskeletal problems, providing evidence for being a more potent option compared with other topical treatments. Despite the effectiveness of comfrey root extract, there are some concerns related to its safety. Comfrey roots contain about 0.2%–0.4% pyrrolizidine alkaloids (PAs): Symphytine, lycopsamine/intermedine (diastereoisomers), acetyl-lycopsamine/acetyl-intermedine (diastereoisomers), myoscorpine, lasiocarpine, heliosupine, viridiflorine, echiumine, symlandine and echimidine [173,174]. PAs may also be present in the comfrey plants as *N*-oxides forms. Both PAs and their *N*-oxides are biologically inactive compounds without any toxicity. However, in the body, they are metabolically activated by hepatic cytochrome P450 (CYPs) drug metabolizing enzymes, specifically CYP3A and CYP2B isoforms [40]. These reactions yield corresponding pyrrolic metabolites that can react with cellular macromolecules, such as proteins and DNA, triggering genotoxic and/or carcinogenic effects [175]. Although internal use of comfrey root extracts is traditionally recommended for rheumatoid arthritis, bronchitis, allergies, diarrhea, gastritis and gastroduodenal ulcers treatment, its efficacy and safety have never been assessed so far in controlled clinical trials.

Moreover, numerous case reports have shown that internal use of comfrey extracts are associated with severe hepatotoxicity particularly cirrhosis and ascites [40]. For this reason, comfrey used internally has not been recommended and was even restricted in the USA and Canada [176]. In most of the European countries such as Germany, France and England, comfrey root is just limited to external use. According to Commission E, the daily exposed dose should not exceed 100 μg PA, with 1,2 unsaturated necine structure, including its N-oxides [176]. Although PAs percutaneous absorption is low [173], the treatment duration should not be longer than 4–6 weeks/year [177] and comfrey preparations application in skin cracked should be avoided. Most of the clinical trials above-mentioned were performed by using fully licensed medicinal products containing comfrey root extract, which is either PA-free or PA-depleted. The use of these processed root extracts has been proved to be safe with numerous controlled trials—the numbers of drug-related adverse reactions were very low and not serious. However, the use of traditional preparations with comfrey root extract may represent a potential health hazard. Since the total PAs concentrations in above-ground plant parts, especially in the leaves, are considerably lower (15–55 μg/g leaf) than the contents of roots (1380–8320 μg/g root), the use of aerial parts of the comfrey can be a safer alternative for self-medication [72].

Overall, as previously reported by Frost et al. in a critical scoping review [3], randomized and non-randomized controlled trials and observational studies have shown clear evidence of comfrey benefits in ankle distortion, back pain, abrasion wounds and osteoarthritis treatment, with few adverse events. Topical application has proven to be safe; however, more rigorous evaluations from clinical trials and observational data are required to substantiate the traditional topical use of *Symphytum* species for musculoskeletal and blunt affections. No or minor adverse effects were reported in these studies. However, it should be noted that most of the controlled clinical trials mentioned in this review were performed on fully licensed PA-depleted or PA-free medicinal products distributed on the markets. Therefore, patients who rely on traditional comfrey preparations should be aware of potential health hazards.

## 7. Conclusions and Future Perspectives 

Current reviews on *Symphytum* species deepen knowledge on evidence for a wide range of indications. However, most of the data are descriptive, with little information on research strategies or bias risks identified in each study. Another aspect related to *Symphytum* species focuses mainly on pyrrolizidine alkaloids toxic potential, though numbers of biologically active polymers, saccharides, phenolic compounds and terpenoids. Nevertheless, the currently available information is not enough to allow a precise evaluation of comfrey risks and potential benefits. However, ancient herbalists used them in a specific way with doses adequacy, as well as the synergistic use more than single plants, and now only pyrrolizidine-free extracts are used in topical preparations. In fact, it should be noted that only PA-depleted or PA-free extracts appear as fully licensed medicinal products. Although clinical trials sustain the folk topical application of *Symphytum* species in musculoskeletal and blunt injuries, with minor adverse effects, *Symphytum* was also still poorly investigated for its antimicrobial potency. Thus, further studies are needed to provide more in-depth data on *Symphytum* species antimicrobial spectrum, including either the single or pooled plant parts at increasing doses, and using distinct extraction solvents, in order to characterize the active natural products both in vitro and in vivo.

## Figures and Tables

**Table 1:** A literature report on the main constitutions of *Symphytum* species from different geographical regions.

Species	Anatomical Part	Main Components	Reference
*S. asperum*	Roots	Intermedine *N*-oxides, lycopsamine *N*-oxides, 7-acetyl symlandine, 7-acetyl symviridine, myoscorpine, symphytine, echimidine	[43,44]
*S. asperum*	Roots	Echimidine, 7- acetyl lycopsamine, 3‘-acetyl lycopsamine, triangularine, and heliosupine	[45]
*S. asperum*	Roots	Anticomplementary dihydroxycinnamate-derived polymer	[46]
*S. asperum* and *S. caucasicum*	Roots	Poly[oxy-1-carboxy-2-(3,4- dihydroxyphenyl)ethylene], poly[3-(3,4-dihydroxyphenyl)glyceric acid	[47]
*S. asperum* and *S. caucasicum*	Roots and stems	Poly[oxy-1-carboxy-2-(3,4-dihydroxyphenyl) ethylene]	[48]
*S. caucasicum* Bieb.	Roots	Asperumine, echimidine *N*-oxide, echinatine and lasiocarpine	[44,49]
*S. cordatum* (L.) W.K	Roots	Echimidine *N*-oxide (three diasteroisomers), 7-sarracinyl-9-viridiflorylretronecine (two diasteroisomers), echimidine (two diasteroisomers), lycopsamine (two diasteroisomers), dihydroechinatine N-oxide, dihydroheliospathuline N-oxide, lycopsamine N-oxide (three diasteroisomers), 7-acetyl lycopsamine *N*-oxide, symphytine *N*-oxide (two diasteroisomers) and 2,3-epoxyechiumine N-oxide	[50]
*S. officinale*	Roots	Intermedine, lycopsamine, intermedine *N*-oxide, lycopsamine *N*-oxide, 7-acetyl intermedine, 7-acetyl lycopsamine, 7- acetyl intermedine *N*-oxide, 7-acetyl lycopsamine *N*-oxide, uplandicine *N*-oxide, myoscorpine, echiumine, symphytine, symviridine, myoscorpine *N*-oxide, echiumine *N*-oxide, symphytine *N*-oxide, symviridine N-oxide, heliosupine, asperumine, heliosupine N-oxide, asperumine *N*-oxide	[51]
*S. officinale*	-	Lycopsamine, echimidine, lasiocarpine	[52]
*S. officinale*	Roots	Symlandine, symphytine, echimidine	[53]
*S. officinale*	Roots	Lycopsamine	[54,55]
*S. officinale*		7-acetyl intermedine, 7-acetyl lycopsamine	[56]
*S. officinale*, *S. asperum, S.× uplandicum*	Roots	Symviridine	[43]
*S. officinale, S. × uplandicum*	Roots	Intermedine, intermedine *N*-oxide, 7-acetyl lycopsamine, acetyl lycopsamine N-oxide, symphytine, symphytine N-oxide, uplandicine, uplandicine *N*-oxide, echimidine, ehimidine N-oxide	[57]
*S. officinale*	Roots	Symphytine, echimidine, isobauerenol, β-sitosterol	[58]
*S. officinale*	Roots, seeds	Allantoin	[39]
*S. officinale* & *S. cordatum*	Shoots and roots	Allantoin, p-hydroxybenzoic acid, hydrocaffeic acid, RA, chlorogenic acid	[59]
*S. officinale*	Roots	RA, isomers of salvianolic acid A, B and C, acetyl intermedine, acetyl lycopsamine and their N-oxides	[41]
*S. officinale*	Roots	Symphytoxide A (triterpenoid saponin)	[60]
*S. officinale*	Callus, leaves, leaf-stalks, ovaries, anthers, and roots	Fructan	[61,62]
*S. officinale*	Roots	Bidesmosidic hederagenin hexasaccharide	[63]
*S. officinale*	Seeds	Γ-linolenic acid	[64]
*S. officinale*	Herb and roots	Caffeic, *p*-coumaric and *m*-hydroxybenzoic acids	[65]
*S. tuberosum*	Whole plant	Anadoline, echimidine	[66]
*S.* × *uplandicum* Nyman (syn. *S. peregrinum* Ledeb.)	Roots and leaves	Intermedine, lycopsamine, 7-acetyl intermedine, 7-acetyl lycopsamine, uplandicine, symlandine, symviridine, myoscorpine, symphytine, echimidine	[44,67]

**Table 2:** Content (ng/g) of pyrrolizidine alkaloids from different parts of *Symphytum officinale* L. [51].

Plant Part	Compounds	Amounts (ng/g)
Roots	Intermedine	280–12,400
Roots	Lycopsamine	800–15,000
Stems	Intermedine	8220
Stems	Lycopsamine	1000
Leaves	Intermedine	160–6600
Leaves	Lycopsamine	180–3400

**Table 3:** Bioactivities of different *Symphytum* species.

Species	Plant Part	Bioactive Compounds	Bioactivity	Effect	Reference
*S. officinalis*	Roots	Phenolic compounds (flavonoids)	Antioxidant	Higher in vitro antioxidant activity than ascorbic acid	[39]
*S. officinalis*	Roots	Phenolic acids	Antioxidant	High antioxidant activity of the extract	[9]
*S. officinalis*	Roots	Poly[3-(3,4-dihydroxyphenyl) glyceric acid]	Antioxidant	Interfere in the formation of active oxygen species	[117]
*S. officinalis*	Roots	Phenolic acids (rosmarinic and caffeic acids)	Antioxidant	Scavenge free radicals, reducing 15-LO inhibition	[41]
*S. officinalis*	Leaves	Phenolic compounds	Antifungal	Inhibit the germination of fungal pathogens	[20]
*S. sylvaticum*	Roots/Leaves	Alkaloid echimidine-*N*-oxide	Antifungal	Strong activity against *Epidermophyton floccosum, Epidermophyton floccosum, Nigrospora oryzae, Allefsheria boydii, Pleuretus ostreatus, Stachbotrys atra, Curvularia lunata* and *Drechslera rostrata*; moderately active against *Aspergillus niger*	[112]
*S. officinalis*	Leaves	NA	Antibacterial	Partial and strong inhibition against mainly *Staphylococcus aureus, Bacillus subtilis, Pseudomonas aeruginosa, Salmonella typhi*	[115]
*S. officinalis*	Roots/Leaves	NA	Antibacterial	Leaves—inhibitory effect against *Bacillus cereus*; Roots—maximum inhibitory effect against *Proteus vulgaris* and *Staphylococcus aureus*	[114]
*S. officinalis*	Roots	Phenolic compounds (ellagic and rosmarinic acids)	Antibacterial	Inhibition against *Escherichia coli* ATCC8739 and *Salmonela typhimirium* ATCC6538	[74]
15-LO, 15-lipoxygenase; NA, Not available information.					

**Table 4:** Summary of the in vitro evidence of *Symphytum* species antimicrobial effects.

Microorganism	Test type	Plant Part	Reference
*Staphylococcus aureus, Bacillus subtilis, Escherichia coli* and *Klebsiella pneumoniae*	Inhibition zone diameter	Aerial propylene glycol extracts	[125]
*S. sclerotiorum*	Antagonistic effect by growth inhibition zones	Endophytic fungi isolated from leaves	[124]
*Staphylococcus aureus; Pseudomonas aeruginosa; Salmonella typhimurium; Shigella sonnei; Klebsiella pneumoniae* and *Escherichia coli*	Inhibition zone diameter more than 7 mm	Leaves extract	[126]
No antimicrobial activity with 34 pathogenic bacterial and fungal isolates	Diameter of the zone of inhibition	Aqueous leaves extract	[116]
*Staphylococcus aureus*	Disc diffusion method	Leaves	[127]
*Staphylococcus aureus*	Disc diffusion method	Ethanolic plant extract	[128]
*Escherichia coli, Salmonella typhimurium*	Minimum inhibitory concentration (MIC)	Aqueous extract of root	[129]
*Bacillus cereus, Proteus vulgaris* and *Staphylococcus aureus*	Disc diffusion method Disc diffusion method	Chloroform leaf extract Methanol root extract	[115]
*Bacillus subtilis* *Staphylococcus aureus, Bacillus subtilis, Pseudomonas aeruginosa, Salmonella typhi*	Streak plate method	Chloroform leaf extract Ethanol leaf extract	[115]
*Bipolaris oryzae*	Antifungal activity by Kirby-Bauer and incubation	Plant aqueous extracts	[123]
*Erysiphe graminis* conidia and *Puccinia graminis* uredospores	Wheat stem	Leaves extract	[20]

**Table 5:** Antioxidant effect of different *Symphytum* species.

Symphytum spp.	Extract or Fraction	Experimental Model	Key Results	Reference
S. asperum roots	Water-soluble hydroxycinnamate-derived polymer	DPPH	0.72 µg/mL	[132]
		Lipid Peroxidation	0.01 µg/mL	
		HR scavenging	≥100 µg/mL	
		SO anion scavenging	13.4 µg/mL	
S. officinale root	Ethanol extract 65%	DPPH	80.25 µg/mL	[41]
		ABTS	20.14 µg/mL	
		FRAP	32.75 µg/mL	
		15-LO	63.68 µg/mL	
S. officinale root	Ethanolic concentrated extract	DPPH	80% inhibition	[134]
S. officinale root	Hot water extraction (25 μg)	ABTS SOD activity	9.61 TE μM 48.98%	[135]
S. officinale L leave	Methanol extract	DPPH	119.96 µg/mL	[136]
		ABTS	193.65 µg/mL	
S. officinale leaves	Ethanolic extract	DPPH	39.97 µg/mL	[77]
		SO scavenging	190.76 µg/mL	
	Aqueous extract	DPPH	96.21 µg/mL	
		SO scavenging	307.42 µg/mL	
S. officinalis roots	Concentrated ethanol extract	DPPH ABTS	374.67 TE µM/g 1152.01 TE µM/g	[8]
	Concentrated methanol extract	DPPH ABTS	207.81 TE µM/g 874.81 TE µM/g	
S. caucasicum leaves	Ethanol extract	DPPH	27.5 µg/mL	[137]
S. officinale root	Ethanol/water extract Concentration 100 μg/ml	DPPH FRAP	0.985 TE mM/g 0.274 AAE mM/g	[9]
	Water extract Concentration 100 μg/ml	DPPH FRAP	0.288 TE mM/g 0.065 AAE mM/g	
S. officinale roots	Water soluble high-molecular-weight biopolymer HP-et (Precipitated by ethanol) Water soluble high-molecular-weight biopolymer HP-ac (Precipitated by acetone)	CL_lum_ OPZ-stimulated PMN	85.4 μg/mL 125 μg/mL	[117]
		CL_luc_ OPZ-stimulated PMN	57.4 μg/mL 88.4 μg/mL	
		CL_luc_ PMA-stimulated PMN	97.8 μg/mL 70 μg/mL	
		CL_luc_ in system HX/XO	1.5 μg/mL 0.7 μg/mL	
S. asperum roots and stems	Roots water soluble fractions from the Stems water-soluble fractions	CL_lum_ (OPZ-activated PMNs)	79.6 μg/mL 113.0 μg/mL	[48]
		CL_luc_ (OPZ-activated PMNs)	82.0 μg/mL 66.5 μg/mL	
		CL_luc_ (PMA-activated PMNs)	74.6 μg/mL 107.4 μg/mL	
		Cl_luc_ (HX/XO system)	2.0 μg/mL 0.75 μg/mL	
S. caucasicum roots and stems	Root Water-soluble fractions Stems Water-soluble fractions	CL_lum_ (OPZ-activated PMNs)	150.5 μg/mL 149.6 μg/mL	[48]
		CL_luc_ (OPZ-activated PMNs)	108.6 μg/mL 113.0 μg/mL	
		CL_luc_ (PMA-activated PMNs	104.5 μg/mL 170.7 μg/mL	
		Cl_luc_ (HX/XO system)	3.2 μg/mL 3.0 μg/mL	
S. asperum leaves	High molecular weight fractions	CL_lum_ OPZ-stimulated PMN	52.0 μg/mL	[10]
		CL_luc_ OPZ-stimulated PMN	27.0 μg/mL	
		CL_luc_ in system HX/XO	1.2 μg/mL	
S. caucasicum leaves	High molecular weight fractions	CL_lum_ OPZ-stimulated PMN	58.0 μg/mL	[10]
		CL_luc_ OPZ-stimulated PMN	31.0 μg/mL	
		CL_luc_ in system HX/XO	1.5 μg/mL	

DPPH, 2,2-diphenyl-1-picrylhydrazyl; AOS, active oxygen species; PMN, polymorphonuclear neutrophils; CL_lum_, luminol-induced chemiluminescence; CL_luc_, lucigenin-induced chemiluminescence; OPZ, opsonized zymosan; FMA, phorbolmyristateacetate; SO, superoxide; SOD, superoxide dismutase; FRAP, ferric reducing ability of plasma; ABTS, 2,2′-azinobis-(3-ethylbenzothiazoline-6-sulfonic acid); HX–XO, Hypoxanthine–xanthine oxidase; HO, Hydroxyl radical; 15-LO, 15-Lipooxygenase; TE, Trolox equivalent; AAE, ascorbic acid equivalent.

**Table 6:** Clinical trials with *Symphytum* species.

Study Design	Treatment	Diagnosis Patients	Key Effects	References
Clinical trial randomized placebo-controlled	Comfrey root-based cream containing mistletoe or placebo Duration: -	OA knee Adult (*n* = 61)	Greater reduction in morning/evening pain (both 28%) and night pain (51%) in the herbal group as compared to placebo	[150]
Clinical trial randomized double-blind bi-center placebo-controlled	Comfrey root extract (1:2, ethanol 60%, *v*/*v*, 35%) Kytta-Salbe^®^ f or placebo Duration: 21 days	OA knee Adult (*n* = 220)	Total score of pain at rest and pain on movement improved by 54.7% in the verum group, 10.7% in the placebo group	[151]
Clinical trial randomized multicenter reference-controlled	Comfrey root-based cream containing tannic acid and eucalyptus (4Jointz) or reference cream with the only *Eucalyptus* Duration: 6 weeks	OA knee Adult (*n* = 43)	Comfrey-based creams more effective in relieving pain, stiffness and improving daily function than the eucalyptus reference cream	[138]
Clinical trial randomized bi-center double-blind placebo-controlled	Comfrey root-based cream containing the tannic acid, aloe vera gel, eucalyptus oil, frankincense oil (4Jointz) or placebo Duration: 12 weeks	OA knee Adult (*n* = 133)	Pain scores significantly reduced in 4Jointz-received group compared to the placebo group	[152]
Clinical trial randomized double-blind multicenter placebo-controlled	Comfrey root extract (1:2, ethanol 60%, *v*/*v*, 35%), Kytta-Salbe^®^f or placebo Duration: 5 days	Back pain Adult (*n* = 120)	Reduction in the intensity of the pain on active standardized movement about 95.2% in the verum group and 37.8% in the placebo group	[153]
Clinical trial randomized multicenter double-blind three-arm placebo-controlled	Comfrey root extract (1:2, ethanol 60%, *v*/*v*, 35%) with 1.2% methyl nicotinate (combination cream: Kytta-Balsam Wf) or reference with only 1.2% methyl nicotinate or placebo Duration: 5 days	Back pain Adult (*n* = 379)	Combination cream and 1.2% methyl nicotinate resulted in statistically significant and clinically relevant reductions in pain scores and increases in tenderness compared to placebo	[154]
Clinical trial randomized pilot study placebo-controlled	Comfrey root ointment or placebo Duration: 4 weeks	Rheumatism (different forms) Adult (*n* = 41)	Clearly better efficacy of comfrey ointment as compared to placebo, regarding "tenderness on pressure" for patients with epicondylitis and tendovaginitis	[155]
Clinical trial randomized double-blind multicenter placebo-controlled	Comfrey root extract (35%), (Kytta-Salbe^®^ f) or placebo Duration: 8 days	Unilateral ankle sprains Adult (*n* = 142)	Comfrey treatment was clinically and significantly superior regarding the reduction of pain and ankle edema, ankle mobility and a global efficacy	[95]
Clinical trial randomized, single-blind multicenter parallel-group	Comfrey root extract (35%), (Kytta-Salbe^®^ f) or diclofenac Duration: 7 days	Unilateral ankle sprains Adult (*n* = 164)	Ankle swelling: Decreased 79.5% in the comfrey root extract group, decreased 69.4% in the diclofenac group; pain on pressure: Reduced 80.6% in the comfrey root extract group, decreased 74.7% in the diclofenac group.	[156]
Open uncontrolled	Comfrey extract obtained from the aerial parts of the plant Duration: 14 days	Painful locomotor system disorder Adult (*n* = 105)	Very effective results in the treatment of subacute and chronic complaints accompanied by functional symptoms like swellings -excellent results in 90%–94 % of patients	[80]
Clinical trial randomized double blind multicenter reference-controlled	10% comfrey extract from the aerial parts of *Symphytum × uplandicum* Nyman (Traumaplant^®^) or reference with 1% active ingredient Duration: 14 days	Ankle distortion Adult (*n* = 203)	Reduction of pain on active motion, pain at rest and functional impairment found to be highly significant, and excellent efficacy in 85.6% of cases with verum compared to 65.7% of cases with reference	[157]
Clinical trial randomized double-blind multicenter reference-controlled	10% comfrey extract from the aerial parts of *Symphytum × uplandicum* Nyman, Traumaplant^®^ or reference with 1% active ingredient Duration: 10 days	Myalgia Adult (*n* = 215)	Pain on active motion, pain at rest and pain on palpation were significantly better reduced in the verum group compared to the reference group, and no systemic adverse effects	[158]
Clinical trial randomized double-blind reference-controlled	10% comfrey extract from the aerial parts of *Symphytum × uplandicum* Nyman, Traumaplant^®^ or reference with 1% active ingredient Duration: 10 days	Fresh abrasions Adult (*n* = 278)	Significant and clinically relevant faster initial reduction of wound size (49% ± 19% versus 29% ± 13% per day), and efficacy in 93.4% of cases of the verum group as compared to reference group (61.7% of cases)	[159]
Open uncontrolled	10% comfrey extract from the aerial parts of *Symphytum × uplandicum* Nyman, Traumaplant^®^ Duration: 9 days	Blunt traumata Children (*n* = 196)	Clear improvement in the range of 84.5% to 100% for every individual parameter such as pain on palpitation, pain in motion, functional impairment, edema and hematoma	[160]
Clinical trial randomized, double-blind reference-controlled	10% comfrey extract from the aerial parts of *Symphytum × uplandicum* Nyman, Traumaplant^®^ or reference with 1% active ingredient Duration: 9 days	Fresh abrasions Children (*n* = 108)	Cream with higher active ingredient concentration reached to 50% healing rate 0.9 days earlier than that of the lower concentration cream	[161]
Pharmacological Trial	5% or 10% of a comfrey root extract (2:7, 50% ethanol) or diclofenac Duration: -	Experimentally-induced UV-B erythema Adult (*n* = 28)	Greater anti-inflammatory potency of the extract than diclofenac (or equal)	[162]
Open uncontrolled	10% comfrey extract from the aerial parts of *Symphytum × uplandicum* Nyman, Traumaplant^®^ Duration: 4 weeks	Decubitus ulcers Adult (*n* = 161)	89.2% reduction in total decubitus area and 88% decrease in depth of the pressure ulcer	[163]
